# Lights and shades of front-line treatment with covalent BTK inhibitors combined with venetoclax in patients with chronic lymphocytic leukemia

**DOI:** 10.3389/or.2025.1703228

**Published:** 2025-12-18

**Authors:** Andrea Visentin, Francesca R. Mauro

**Affiliations:** 1 Hematology Unit, Department of Medicine, University of Padua, Padua, Italy; 2 Hematology, Department of Translational and Precision Medicine, Sapienza University of Rome, Rome, Italy

**Keywords:** acalabrutinib, chronic lymphocytic leukemia, fixed duration therapy, ibrutinib, venetoclax, zanubrutinib

## Abstract

This review focused on trials investigating the front-line covalent BTK inhibitors (cBTKi) and venetoclax (V) combination administered in a fixed-duration (CAPTIVATE, GLOW, AMPLIFY trials) or minimal residual disease (MRD)-guided manner (MDACC, FLAIR, ERADIC, HOVON NEXT STEP, SEQUOIA arm D trials) in patients with chronic lymphocytic leukemia (CLL). We reviewed data from these studies to highlight their therapeutic activity and toxicity profile. Despite the heterogeneity in patients’ characteristics, treatment schedule, and duration, most patients achieved deep responses with undetectable MRD and prolonged progression-free survival (PFS) with BTKi + V regimens. MRD-guided treatments yielded higher PFS rates, including patients with high-risk genetic characteristics, such as those with unmutated IGHV and *TP*53 disruption. The cBTKi + V regimen is easy to manage and relatively well tolerated. However, cBTKi-related cardiovascular toxicities remain a limiting concern, especially for the use of cBTKi + V in some older patients.

## 1 Introduction

Inhibitors of the Bruton’s Tyrosine Kinase (BTK) and BCL2 (B-cell lymphoma 2) are two highly effective classes of agents in the management of patients with chronic lymphocytic leukemia (CLL) either as single agents or in combination.

Several studies have demonstrated the increased efficacy of these agents when used in combination, due to their synergistic effects resulting from complementary mechanisms of action (1).

These drugs target distinct but interrelated pathways that promote the survival of chronic lymphocytic leukemia (CLL) cells.

Ibrutinib (I) is the first-in-class covalent BTK inhibitor (cBTKi), which disrupts the B-cell receptor (BCR) signaling pathway, thereby inhibiting the survival and proliferation of CLL cells.

**FIGURE 1:**
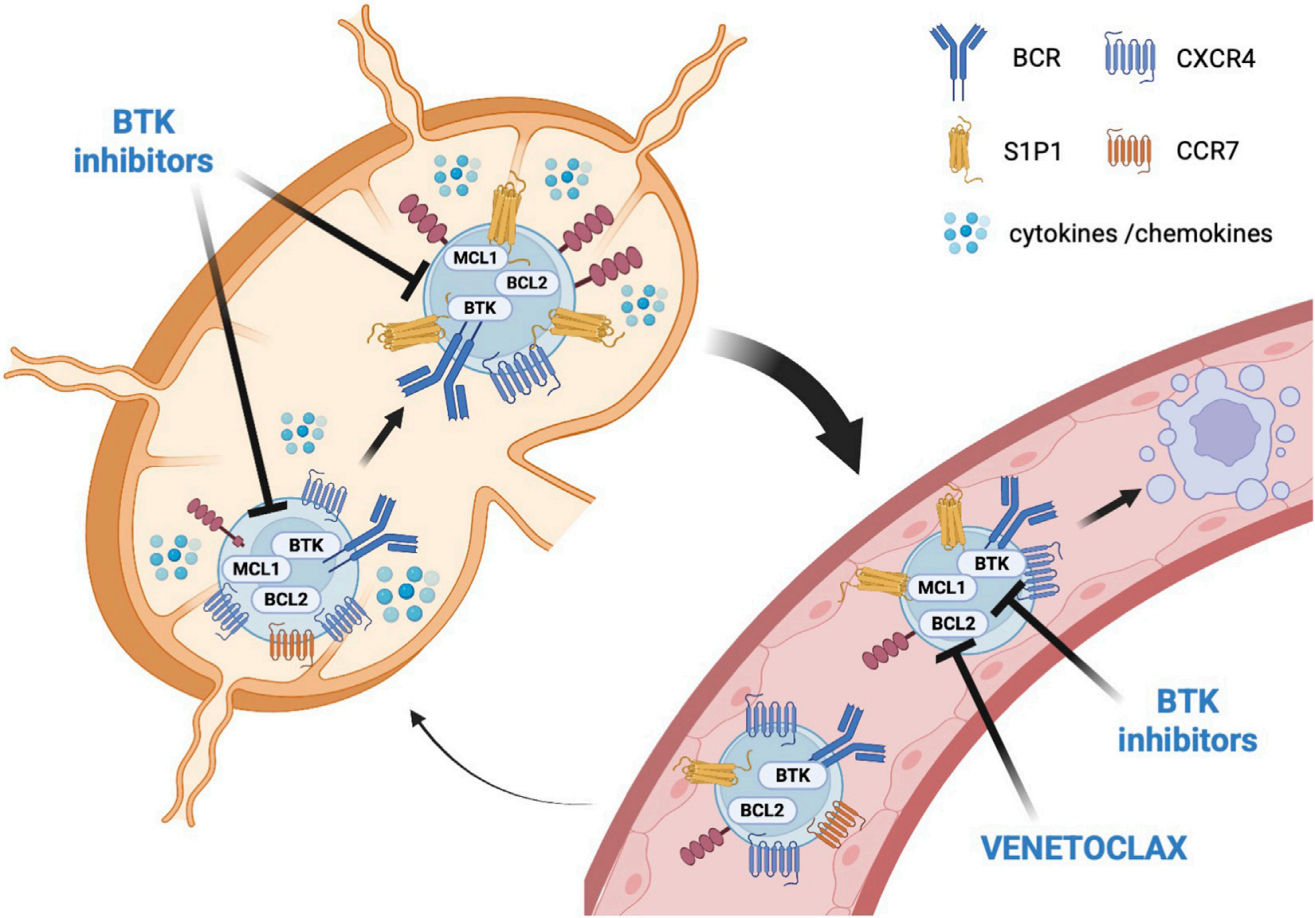
Mechanisms exerted by BTKi (ibrutinib or acalabrutinib or zanubrutinib) and venetoclax individually and synergistically on the factors involved in the homing and apoptosis of leukemic CLL cells. While the synergism of ibrutinib and venetoclax have ben extensive studied, there are few studies of acalabrutinib + venetoclax and zanubrutinib + venetoclax.

Moreover, I downregulates the homing receptors CXCR4 and CCR7 and upregulates egress receptors such as S1P1 (2, 3) (Figure 1). By disrupting the adhesion of CLL cells to the protective microenvironment of lymph nodes and bone marrow, I facilitates the mobilization and migration of these cells into the peripheral blood. In addition, TLR9 stimulation in the lymph node microenvironment increases CD40 expression, favoring the expression of BCL2 and other anti-apoptotic proteins that mediate *in vitro* resistance to venetoclax (4).

Venetoclax (V) is a BCL-2 inhibitor that targets an anti-apoptotic protein overexpressed in CLL cells, helping them evade programmed cell death. By inhibiting BCL-2, V restores the apoptotic pathway, leading to the rapid death of CLL cells. V is particularly effective against CLL cells in the peripheral blood, where their mobilization due to I makes them less protected by survival signals from the microenvironment and more accessible to venetoclax (4) (Figure 1).

It has also been demonstrated that I and V act on distinct CLL subpopulations with different proliferative capabilities. The proliferative subpopulation (CXCR4dim CD5bright) of CLL cells is more sensitive to I. In contrast, the resting subpopulation (CXCR4bright CD5dim) preferentially responds to V (5) (Figure 1). Consequently, the combination of I + V effectively targets both the resting and dividing CLL subpopulations.

In addition to the complementary actions on CLL biology, preclinical models have suggested that the combination of I + V may be synergistic. Specifically, the inhibition of BTK by I results in reduced levels of myeloid cell leukemia 1 (MCL1) protein. By suppressing MCL-1, I increases the dependency of CLL cells on BCL-2, making them more vulnerable to the apoptotic effects of V, thereby creating a synergistic effect (6) (Figure 1).

In particular, Deng J. et al. (7) studied the cell’s proximity to the apoptotic threshold by BH3 profiling, showing that both ibrutinib and acalabrutinib increase the CLL cell’s dependence on BCL-2, thereby enhancing the sensitivity of BCL2 inhibition. Consistently, *ex vivo* treatment with BTKi increases the protein expression of BIM, a pro-apoptotic protein. In addition, *ex vivo* exposure to ibrutinib, but not acalabrutinib, decreases cell viability after cBTKi + V. No preclinical study is available with zanubrutinib and BCL2 inhibitors.

Given their complementary clinical activities resulting from the dual targeting of different survival pathways, the preclinical evidence of synergy, and their non-overlapping toxicities, the cBTKi + V combination has been extensively studied for treating CLL (8–12).

Clinical trials have demonstrated that these combinations result in high rates of CR and undetectable measurable residual disease (uMRD) in treatment-naïve patients. Deep responses have been detected even in those patients with *TP*53 abnormalities (del (17p) and/or *TP*53 mutation) and/or complex karyotype (CK, defined by the presence of at least 3 cytogenetic abnormalities) (9, 10, 12).

The achievement of deep responses has enabled fixed-duration treatment approaches, which reduce long-term exposure to toxicities and enhance patient convenience compared to indefinite therapies. Furthermore, the strategic lead-in phase with cBTKi improves safety by reducing the leukemic burden and the risk of tumor lysis syndrome (TLS), allowing for safer administration of venetoclax (11, 13). The efficacy of the cBTKi + V combination has also been investigated in an MRD-guided manner, extending treatment until a deep response with undetectable MRD is achieved (12, 14, 15). This personalized treatment modality adapts to the specific characteristics of each patient’s disease, which is promising, but further validation through prospective trials is necessary.

Our observational, descriptive study focused on trials investigating the efficacy and safety of treatments combining cBTKi and V, administered either as fixed-duration or MRD-guided therapy. The descriptive data we present can highlight the critical points and strengths of the cBTKi + V combination in both treatment modalities.

## 2 Patients and methods

This review aims to provide an overview of the efficacy and safety of the front-line cBTKi + V combination given as first-line treatment in patients with CLL. We analyzed published, peer-reviewed papers till June 2025. The studies we evaluated included prospective trials that enrolled patients with CLL treated with I + V. The search was conducted using the PUBMED database with the keywords “chronic lymphocytic leukemia” AND “venetoclax” OR “ibrutinib” OR “acalabrutinib” OR “zanubrutinib”. Additionally, a manual search was performed using the same keywords in materials from the EHA, ASCO, and ASH congresses. This process resulted in identifying 7,931 records for studies published up to July 2025. We only included prospective clinical trials reported in English, that involved at least 50 patients. We excluded meta-analyses, pooled analyses, retrospective studies, guidelines, comments, observational studies, case reports, and letters from this review. When multiple updates of the same study were available, we only considered the most updated. We selected 9 studies that met these criteria. The PRISMA flow diagram reported in Supplementary Figure S1 describes our selection process.

The primary endpoint of this review was to describe the efficacy of the cBTKi + V regimens in terms of response rates (overall response rate ORR; CR rate according to the iwCLL guidelines (16) and the rate of responses with undetectable MRD (uMRD: residual leukemic cells: <10^–4^) in the peripheral blood (PB) and/or bone marrow (BM). We also considered the survival rates of each trial in terms of progression-free survival (PFS) and overall survival (OS).

The secondary endpoints included the description of treatment duration, the median follow-up for each study, and the clinical and biological characteristics of the enrolled patients. We also considered the rates of patients with AEs (AEs), including neutropenia, infections, bleeding events, cardiovascular events, hypertension, and atrial fibrillation, as per CTACE 5.0. Rates of treatment discontinuations due to AEs and sudden/unexplained deaths were also considered.

## 3 Results

The clinical and biological characteristics of patients enrolled in the various trials that investigated cBTKI + V regimens along with their outcomes are summarized in Table 1. Table 2 reports the survival outcomes of patients categorized by the IGHV mutational status and the presence of *TP*53 aberrations. Table 3 summarizes the rates of AEs of interest reported in in the different trials.

**TABLE 1:** Efficacy results from front-line clinical trials investigating ibrutinib plus venetoclax in patients with CLL.

Trials	Duration of I + V (months)	Phase	Comparator	N	Age (y)	Median FUP (months)	% OR/CR	% uMRD4^a^ PB/BM	% PFS	% OS
*Trials investigating fixed-duration I + V*										
*FD-cohort CAPTIVATE* (10, 17, 18)	15	2	Single arm	159	60	28	96/55	77/60^f^	5.5y: 60	5y: 96
*MRD-cohort CAPTIVATE* (15)	15	2	MRD arm	164	58	31	97/46	75/68^f^	NR	NR
*GLOW* (8, 19, 20)	15	3	G-Chl	106	71	57	87/39	61/56^f^	5.5y: 52	5.5y: 79
*Trials investigating MRD-guided I + V*										
*MDACC*° (21, 22)	24–36	2	Single arm	120	64.5	61.5	81/69	NR/64^f^	5y: 90	5y: 96
*FLAIR* (14)	24–60	3	FCR, ibrutinib	260	62	44	84/71	2y 73.1/66.2^f^	5y: 93.9	3y: 98
*NEXT STEP* ^b^ (25)	15	3	Single arm	84	69	35	94/40	52/43^f^	2y: 94	2y: 98
*ERADIC* (23)	15–27	2	FCR	60	61	42	88/68	85/68^f^	2.3y: 95	2.3y: 95
*Trials investigating fixed-duration A + V*										
*AMPLIFY* (11)	14	3	AVO, FCR/BR	291	60	41	93/9	45/40^f^	3y: 77	3y: 94
*Trials investigating MRD guided Z + V*										
*SEQUOIA arm D* (12)	≥28	2	Single arm	114	67	31.2	97/48	59/48^f^	2y: 92	2y: 96

Abbreviations: N, number; ORR, overall response rate; CR, complete response; PFS, progression-free survival; OS, overall survival; FD, fixed-duration; uMRD, undetectable measurable residual disease; PB, peripheral blood; BM, bone marrow; FUP, follow-up; NR, not reported; G-CHL, obinutuzumab-chlorambucil; y, years; M, median; I + V, ibrutinib plus venetoclax; A + V, acalabrutinib plus venetoclax; Z + V, zanubrutinib plus venetoclax.

^a^ Method to assess MRD: f, flow-cytometry.

^b^ The data reported refer to the response assessed after the end of the combination treatment with ibrutinib and venetoclax (I + V). Survival rate are not described as they are impacted by further treatments after randomization.

^(°)^ The rates of overall response and complete responses are calculated on 80 patients based on Jain N et al, JAMA, 2021.

**TABLE 2:** Efficacy results from frontline clinical trials investigating ibrutinib plus venetoclax according to the IGHV mutational status and *TP*53. abnormalities.

Trials	Duration of I + V (months)	N IGHV	U/M ratio	% with uMRD (10^–4^) PB/BM^a^	% PFS	% OS	N with TP53 abn.	% with uMRD4 PB/BM^a^	% PFS	% OS
*Trials investigating fixed-duration I + V*										
*FD-cohort CAPTIVATE* (10, 17, 18)	1	U: 89	1.34	84/64	5.5y: 53	5y: 93	Yes: 27	81/41^f^	5.5y: 30	5y: 90
		M: 66		67/53	5.5y: 80	5y: 100	No: 81	76/62^f^	5.5y: 60	5y: 100
*MRD cohort* ^b^ *CAPTIVATE* (15)	15	U: 99	1.57	NR/77	NR	NR	Yes:32	NR/66^f^	NR	NR
		M: 63		NR/56	NR	NR	No: 120	NR/72^f^	NR	NR
*GLOW* (8, 19, 20)	15	U: 67	2.09	60/NR	5.5y: 41.6	5.5y: 76.5	Yes: 7	NR	NR	NR
		M: 32		41/NR	5.5y: 76.4	5.5y: 89.8	No: 99	NR	NR	NR
*Trials investigating MRD-guided I + V*										
*MDACC*° (21, 22)	24–36	U: 103	4.88	NR/67	3-y: 90	NR	Yes: 28	NR/77^f^	5-y 86.1	NR
		M: 17		NR/54	NR	NR	No: 92	NR/NR	3-y 92	NR
*FLAIR* (14)	24–60	U: 123	6.05	95/72	5-y: 94.9	3y: 99.2	Yes: 1	NR/NR	NR	NR
		M: 93		72/60	5-y: 90.1	3y: 95.5	No: 259	NR/NR	NR	NR
*NEXT STEP* (25)	15	U: 37	1.32	NR/NR	NR	NR	Yes: 6	NR/NR	NR	NR
		M: 41		NR/NR	NR	NR	No: 78	NR/NR	NR	NR
*ERADIC* (23)	15–27	U: 60	0.90	85/68	2.3y: 95	2.3y: 100	Yes: 0	—	—	—
		M: 0		—	—	—	No: 60	85/68^f^	27m: 95	27m: 100
*Trials investigating fixed-duration A + V*										
AMPLIFY (11)	14	U: 167	1.35	54/NR	3y: 69	NR	Yes: 0	—	—	—
		M: 124		33/NR	3y: 86	NR	No: 291	45/40^f^	3y: 77	3y: 94
*Trials investigating MRD guided Z + V*										
*SEQUOIA arm D* (12)		U: 86	3.6	NR	2y: NR	NR	Yes: 66	59^f^/NR	2y: 94	2y: NR
		M: 24		NR	2y: NR	NR	No: 47	60^f^/NR	2y: 89	2y: NR

Abbreviations: N, number; ORR, overall response rate; CR, complete response; PFS, progression-free survival; OS, overall survival; FD, fixed-duration; uMRD, undetectable measurable residual disease; uMRD4, less than one chronic lymphocytic leukemia cell per 10,000 leukocytes (<10–4); PB, peripheral blood; BM, bone marrow; FUP, follow-up; NR., not reported; G-CHL, obinutuzumab-chlorambucil; y, year; m, month. I + V, ibrutinib plus venetoclax; A + V, acalabrutinib plus venetoclax; Z + V, zanubrutinib plus venetoclax.

^a^ Method assessed to assess MRD: f, flow-cytometry; n, NGS.

^b^ The data reported refer to the response assessed after the end of the combination treatment with ibrutinib and venetoclax (IV). The PFS, and OS, data are not described as they are impacted by the subsequent therapeutic approach.

^(°)^ Data referred to an interim analysis on 80 patients. MRD, data referred to the assessment at 24 months.

**TABLE 3:** Safety results from frontline clinical trials investigating ibrutinib plus venetoclax in patients with CLL.

Trials	Age (y)	I + V duration	% TD AE	% G ≥ 3 neutropenia	% G ≥ 3 infections	% G ≥ 3 bleedings	% G ≥ 3 CV	% G ≥ 1 HTN	% G ≥ 1 AF	% TLS^(^°^)^	N sudden/unexplained deaths
*Trials investigating fixed-duration I + V*											
*FD-cohort CAPTIVATE* (10, 17, 18)	60	15	4.4/0.6	33	8	2	7	6	4	0.0	1
*MRD-cohort CAPTIVATE* (15)	58	15	6/4	35	9	1	4	16	7	0.6	2^a^
*GLOW* (8, 19, 20)	71	15	14	35	17	<5	16	13	14	0.0	4
*Trials investigating MRD-guided I + V*											
*MDACC°* (21, 22)	65	24–36	14/10	51	22	0	20	10	10	4	1
*FLAIR* (14)	62	24–60	23	10	21	2	11	13.5	13.5	6	3
*FLAIR* (14)	66	15	7	NR	25	2	NR	NR	15	NR	0
*ERADIC* (20)	61	15–27	6.7/6.7	3	24	NR	18	NR	6	15	5
*Trials investigating fixed-duration A + V*											
*AMPLIFY* (11)	60	14	7.9	32	12	1	5	4	1	0.3	0
*Trials investigating MRD guided Z + V*											
*SEQUOIA arm D* (12)	67	≥28	25	17	12^b^	4	12	13	3	1	0

Abbreviations: TD AE, treatment discontinuation due to adverse events; I + V, ibrutinib plus venetoclax; A + V, acalabrutinib plus venetoclax; Z + V, zanubrutinib plus venetoclax; CV, cardiovascular events; HTN, hypertension; TLS, tumor lysis syndrome; NR, not reported.

^(°)^ TLS, laboratory cases reported, except for 1 case in the FLAIR, trial.

^a^ Cardiac arrest in 2 patients.

^b^ COVID-19, 2%.

## 4 Trials investigating regimens with fixed-duration I + V

### 4.1 CAPTIVATE trial

The phase II CAPTIVATE trial investigated the I + V combination as first line therapy (3 cycles of ibrutinib followed by 12 cycles of combined I + V) in patients <70 years. This study included an MRD-guided (MRD cohort) and a Fixed Duration (FD cohort) (10, 15). The results of the MRD cohort are described in this section because they refer to the I + V combination phase only. Briefly, the median age of patients was 58 years (range 28–69), 60% of patients had unmutated IGHV gene, 20% *TP*53 abnormalities, and 19% CK. Among the 164 patients included in this trial, the best rates of responses with undetectable MRD (uMRD) after 12 cycles of I + V in the PB and in the BM, were 75% and 68%, respectively. At the end of treatment, patients who reached uMRD were randomized to receive ibrutinib or placebo, while those with detectable MRD (dMRD) were to receive ibrutinib or I + V. With a median follow-up of 31 months the estimated 30-month PFS rate was above 95% in both uMRD and dMRD subgroups. Additional cycles of either ibrutinib (from 32% to 42%) or I + V (from 31% to 66%) allow for an increase in the uMRD rates in the BM. Grade 3–4 neutropenia was recorded in 35% of patients, but severe infections in 9%. Any grade hypertension or atrial fibrillation was recorded in 60% and 7%, respectively, but severe cardiovascular events were seen only in 4%.

One hundred fifty-nine patients were enrolled in the fixed-duration (FD) cohort of the CAPTIVATE trial (10). The median age of patients was 60 years (range 33–71). Fifty-six % of the patients had unmutated IGHV, 19% a complex karyotype (CK), and 17% *TP*53 abnormalities. The CR rate was 55%, and the best uMRD rates were 77% in the PB and 60% in the BM. The updated 5.5-year PFS and OS rates presented at the 2025 EHA meeting were 60%, and 96%, respectively. For IGHV mutated and unmutated patients without *TP*53 disruption 5.5-year PFS rates were 80% and 53%, respectively. A lower 5.5-year PFS rate was reported for patients with *TP*53 disruption (17). Of note, the presence of lymph nodes larger than 5 cm was not associated with lower uMRD rates or shorter PFS (18). The most common grade 3 or higher AEs were neutropenia (33%) and hypertension (6%). Any grade of atrial fibrillation occurred in 4% of patients. While minor bleedings were common, at similar rates in patients receiving or not concomitant anticoagulants/anti-platelets, major hemorrhage occurred in only 2% of patients (10). Severe grade 3 or higher infections were observed in 8% of patients. Notably, no clinical TLS events were reported.

### 4.2 GLOW trial

The GLOW trial is a phase phase 3, randomized, multicenter study that included patients over 65 years old or younger but with comorbidities, defined by a CIRS score above six 6 or a creatinine clearance of less than 70 mL/min.

The median age of patients was 71 years, 51.9% had unmutated IGHV, and 7% had *TP*53 mutation. The best uMRD rates assessed by flow cytometry in the PB and BM were 61% and 56%, respectively, higher than those recorded in patients treated with G-CHL. Compared to mutated IGHV patients, those with unmutated IGHV achieved higher uMRD rates (60% vs. 41%). However, the 3-year-uMRD rate was lower among unmutated than mutated IGHV cases (31% vs. 34%) (19). The 5.5-year PFS and OS rates for patients treated with I + V were 52% and 79%, respectively. IGHV mutation significantly impacted on PFS and OS. After a median follow-up of 67 months, the cumulative rate of patients who required subsequent treatment for mutated and unmutated IGHV patients was 19.7% and 22.8%, respectively (20). Grade 3 or higher AEs were recorded in 76% of patients receiving I + V, similar to G-CHL (70%). Severe neutropenia occurred in 35% of the patients, but grade 3 or higher infections were reported in only half of them. Notably, almost one out of six patients developed hypertension,r atrial fibrillation or a severe cardiovascular event. Four cases of sudden or unexplained deaths were recorded. Additionally, patients experienced a shorter duration in the grade 3/4 treatment-related-AEs and in the progressive disease health state when receiving I + V than the control regimen. It has also been found that PFS time without grade 3/4 toxicity or relapse was extended by 21.4 months for the I + V group (51.6 months) compared to the chlorambucil and obinutuzumab group (30.2 months; p = 0.0052) (20).

## 5 Trials investigating a MRD-guided duration of the I + V regimen

### 5.1 MD anderson cancer center trial

The phase 2 study from the MD Anderson Cancer Center by Jain et al. investigated the I + V combination in patients 65 years or older or with high-risk characteristics such as *TP*53 abnormalities, deletion (11q),and unmutated IGHV (9). The I + V regimen in the original trial was administered for 24 cycles, and patients with persisting residual disease in the BM at the end of treatment continued therapy with I monotherapy. Subsequently, the trial protocol was amended, to allow 12 additional cycles of I + V for patients with dMRD (21). The median age of the 120 patients included in this study was 64.5 years (range 26–88). Unmutated IGHV was present in 86% of patients, and *TP*53 abnormalities in 23% (19). Seventy-seven (64%) patients achieved uMRD in BM after 24 treatment cycles. Responses were seen across all high-risk subgroups (21). After a median follow-up of 61 months, the 5-year PFS and OS rates were 90% and 96%, respectively (22). Among the 24 patients with dMRD in the BM, one patient had Richter transformation while the remaining 23 continued I. After the protocol amendment, 18/23 patients received 12 additional cycles of I + V, and 11/18 (61%) achieved uMRD during the third year of treatment. The safety profile was similar to that described in the Glow trial. However, due to the most extended treatment duration, a higher rate of grade 3 neutropenia (51%), infections (22%), and cardiovascular events (20%) occurred. Four TLS, events and 1 sudden death were recorded.

### 5.2 The FLAIR trial

The FLAIR trial is a phase 3, multicenter, randomized, controlled trial comparing the I + V with the fludarabine-cyclophosphamide-rituximab chemoimmunotherapy (FCR) in patients younger than 70 years and with less than 20% del (17p) at FISH analysis (14). Patients received the I + V combination after 2 cycles of an ibrutinib lead-in phase., then I + V from a minimum of 2 years up to 6 years depending on the time they needed to achieve a response with uMRD in BM. A total of 260 patients were randomly assigned to receive I + V. The median age of patients was 62 years, 50% of them had unmutated IGHV gene, 10% harbored BCR stereotype #2, and 1 patient had del (17p) in <20% of cells.

The rate of patients treated with I + V who achieved uMRD in the BM at 2 years was 52% and rose to 66% at 5 years. IGHV unmutated patients showed higher rates of uMRD (PB: 95%; BM: 72%) than mutated IGHV patients (PB, 72%; BM, 60%). Moreover, 58% of patients who achieved uMRD in BM at 3 years stopped I + V. After a median follow-up of 44 months, the 3-year PFS and OS with I + V were 97% and 98%, respectively, higher than those observed with FCR, 77% and 93%^,^ respectively. The updated results of the FLAIR trial presented at the 2025 EHA meeting showed that for patients who received MRD-guided I + V a significantly higher 5-year PFS (p > 0.001) than those treated with FCR or continuous I, 93.9%, 58.1% and 79%, respectively (14). The same advantage was observed also in OS rates. A higher PFS rate also emerged when unmutated IGHV patients were analyzed. PFS rates for patients treated with MRD-guided I + V, FCR and continuous I, were 94.9%, 49.7%, and 79.9%, respectively. In the unmutated IGHV subset, MRD-guided I + V maintained superiority over patients treated with FCR. The 2-year uMRD rate for patients who received MRD-guided I + V and FCR were, 66.2% and 48.3%, respectively.

In patients receiving MRD-guided I + V the treatment discontinuation rate due to AEs was relatively high, at 23%. The most common grade 3–4 AEs were infections (21%) and cardiovascular events (11%). Any grade of hypertension and atrial fibrillation were both recorded by 13.5% of patients. Sudden deaths were recorded in 3 patients.

### 5.3 ERADIC trial

The ERADIC phase 2 trial included fit patients with CLL with an intermediate risk defined by the presence of unmutated IGHV, del (11q), or CK, but without *TP*53 abnormalities (23). This trial also compared I + V, which was given with an MRD-guided approach to the FCR regimen. After a lead-in phase of 3 cycles of I, patients received the I + V combination, the duration of which was based on MRD assessed on BM at cycle 9. In patients who achieved uMRD in BM at month 9, treatment was continued with I + V for 6 additional cycles (i.e., overall treatment duration 15 cycles), while in those with dMRD, I + V treatment was continued till cycle 27 and then stopped. One hundred and twenty patients were randomized 1:1 between the 2 treatment arms. The patient’s characteristics were well balanced between the two arms. The median age of patients who received I + V was 61 years (range 34–74). All patients but one had unmutated IGHV, and 24% del (11q). At cycle 9, 38% of patients achieved uMRD in BM and 57% in PB (24). The rate of patients, who at cycle 27, reached uMRD in BM and PB increased to 68% and 85%, respectively. With a median follow-up of 42 months, the 2.3-year PFS was significantly higher for the I + V arm compared with the FCR arm (95% vs. 78.5%, p = 0.029), without difference in OS between the two arms (95% for both).

### 5.4 The HOVON NEXT STEP trial

The HOVON NEXT STEP trial is a phase 2 study that enrolled previously untreated patients with CLL without specific genetic characteristics requiring therapy (25). Patients received 3 cycles of I followed by 12 cycles of the I + V combination. While patients achieving CR with uMRD in the BM stopped treatment, those with dMRD received 6 additional cycles of the I+ obinutuzumab (I + O) combination. Eighty-four patients were included in this study with a median age of 68 years (range 59–74), and 8% were diagnosed with SLL. Forty-four % of patients showed an unmutated IGHV status, 8% a CK, and 7% *TP*53 abnormalities. After cycle 15, uMRD was achieved by 44% of patients in the PB and 36% in the BM. Twenty % of the patients achieved a CR with uMRD in the BM and stopped treatment. After I + O intensification, 60% of patients who did not obtain uMRD or CR with I + V reached CR with uMRD in the BM. After a median follow-up of 35 months, the overall 24-month PFS and OS were 94% and 98%, respectively. During the I + V treatment, 75% of the patients developed grade 3 or higher AEs, leading to discontinuation in 7% with only one fatal event (Table 3). Severe infection occurred in 25% of patients and atrial fibrillation in 15%.

## 6 Trial investigating the fixed duration AV regimen

The AMPLIFY trial is a phase 3 multicenter study (11) that included patients over 18 years old without *TP*53 abnormalities. Patients were randomized to acalabrutinib (A) plus venetoclax (A + V) (n = 291), or the A + V + obinutuzumab triplet regimen (AVO, n = 286) or, chemoimmunotherapy (FCR or BR, n = 290). The median age of patients was 60 years including 27% patients 65 years or older; 57.4% had unmutated IGHV. After 14 months of the A + V regimen (2 lead-in months of A and 12 of the A + V combination) the best uMRD rates assessed by flow cytometry in the PB and BM were 45% and 40%, respectively (11). After a median follow-up of 41 months, the 3-year PFS and OS rates were higher with A + V was than with chemoimmunotherapy (77% and 94% vs. 66.5% and 86%) (Table 1). Additionally, patients with unmutated IGHV achieved higher uMRD rates with A + V, (54% vs. 33%) but lower PFS (69% vs. 86%) than IGHV mutated cases (Table 2). Grade 3 or higher AEs were recorded in almost half of patients receiving A + V but at a lower rates compared to chemoimmunotherapy or AVO (69%). Severe neutropenia occurred in 32% of the patients but grade ≥3 infections were experienced by 12% of the patients (Table 3). Notably, very few patients developed hypertension (4.1%), atrial fibrillation (0.7%) or severe cardiovascular events (1.7%). No cases of sudden or unexplained death have been recorded during treatment.

## 7 Trial investigating the zanubrutinib and venetoclax combination

The arm D of the front-line SEQUOIA trial was a non-randomized, MRD-guided study that included patients aged 65 years or younger with comorbidities with or without *TP*53-aberrant disease (12).

Treatment began with the cBTKi zanubrutinib (Z) starting from cycle 1, combined with V (Z + V) from cycle 4 through cycle 28. Patients with uMRD.were eligible for early discontinuation of V after receiving a minimum of 12 cycles or for discontinuation of Z after receiving a minimum of 27 cycles. Treatment with Z continued in patients with dMRD until progressive disease, unacceptable toxicity, or uMRD. This study had a median follow-up of 31.2 months and included 114 patients. Among them, 66 (58%) had *TP*53 abnormalities, 47 (41%) had no *TP*53 abnormalities, and one patient had missing *TP*53 results. The ORR was 97% (CR/CRi: 48%) in the intent-to-treat (ITT) population, 99% (CR/CRi: 47%) in patients with *TP*53-aberrant disease, and 96% (CR/CRi: 49%) in patients without *TP*53-aberrant disease. In the intention-to-treat (ITT) population, 59% of patients achieved PB-uMRD at any point during the study. The PB-uMRD rate for patients with *TP*53-aberrant disease was 59%, while it was 60% for those without *TP*53-aberrant disease. The median time to first detection of PB-uMRD was 19 months for patients with *TP*53 aberrations and 11 months for those without. Of the 11 patients who stopped treatment due to uMRD-guided criteria, nine stayed in clinical remission with sustained PB-uMRD, and one discontinued study while in remission. The 24-month PFS rate was 92%, 94% for patients with *TP*53-aberrant disease, and 89% for those without *TP*53 aberrations. The estimated 24-month OS was 96%

The three most common treatment-emergent AEs of any grade were COVID-19, occurring in 54% of patients, diarrhea reported in 41%, and contusion in 32%. Among the grade ≥3 treatment-emergent AEs, the most frequent were neutropenia (23%), hypertension (10%), and diarrhea (6%). Atrial fibrillation was diagnosed in 3% of patients. Additionally, 29 patients (25%) discontinued Z due to an AE.

## 8 Discussion

This survey aimed to evaluate the strengths and weaknesses of different therapeutic regimens that included V as a backbone, in combination with a cBTKi.

Indirect comparisons of trial results regarding efficacy and safety can be misleading due to bias. Additionally, the significant differences between trials arising from patient inclusion criteria—such as age, fitness level, comorbidities, genetic profiles, treatment duration, and follow-up—limit indirect comparisons and meta-analyses.

Thus, the descriptive data we present can only highlight some strengths and critical points of both fixed-duration and MRD-guided treatment with the different cBTKi + V regimens.

### 8.1 Patients’ characteristics

One finding consistent across cBTKi + V studies, except for the GLOW study, which enrolled patients with a median age of 71 years, is the relatively young age of patients who received these combinations, ranging from 58 to 68 years, which is lower than that reported for patients diagnosed with CLL (26). Notably, due to different inclusion criteria, there was also high variability in the rates of patients with unmutated IGHV and/or *TP*53 abnormalities. Unmutated/mutated ratios ranged between 0.9 and 6.05. Moreover, patients with a disruption of the *TP*53 gene were only included in the CAPTIVATE, MDACC, and SEQUOIA arm D studies (9, 10, 12, 15). In addition, trials show differences in the method used to assess MRD, which was flow cytometry in some studies and next-generation sequencing in others. Most importantly, due to the different study designs, varying durations of BTKi + V treatment have been considered. In the FD-CAPTIVATE GLOW studies (8, 10), the cBTKi + V regimen had a fixed duration of 15 months, whereas in the AMPLIFY A + V treatment it takes 14 months (11). Conversely, in studies utilizing an MRD-guided approach, the length of therapy was based on the depth of response, with treatment durations extended up to 6 years, as seen in the FLAIR study (12, 14, 15, 21, 23, 25).

### 8.2 Rates of responses with uMRD

Despite the heterogeneity in patient characteristics, treatment duration, and follow-up, longer treatment duration seems to be associated with higher rates of uMRD in the BM. At the end of the 12- month FD treatment with A + V (AMPLIFY trial), the uMRD rate in the BM was 40% while it was around 60% with I + V (FD CAPTIVATE and GLOW trials) (8, 10),11. In the MDACC trial, where treatment duration was longer than 24 months and tailored to the specific disease dynamics of each patient, the I + V combination achieved an uMRD rate in the BM of 67% at 27 months (21). A similar 2-year rate of patients achieving uMRD in the BM was reported at 66% in the FLAIR trial, where the I + V combination was administered for up to 6 years (14). Interestingly, a trend toward higher uMRD rates was recorded among patients with unmutated IGHV compared to those with mutated IGHV (10, 14, 15, 19, 21, 27). In addition, a faster time to reach uMRD was observed in patients with unmutated IGHV compared to those with mutated IGHV (27).

The high number of patients who experienced SARS-CoV2 infection in the AMPLIFY trial may justify why the A + V regimen did not achieve the same uMRD rates as those obtained with other cBTK + V regimens (45% in the PB and 40% in the BM) (11).

Deep responses with uMRD have also been observed in patients with *TP*53 disruption treated with I + V in the FD Captivate trial (41%), but with higher rates in trials with an MRD-guided approach such as the MDACC trial (77%) and arm D of the SEQUOIA trial with Z + V (59%) (10, 12, 21).

### 8.3 Progression-free survival

While FD and MRD-guided I + V regimens resulted in similar overall uMRD rates, differences emerged in PFS, with a superior PFS rates in the MDACC and FLAIR trials (5-year PFS rates of 90% and 93.9%, respectively) compared to that observed with FD-I + V in the CAPTIVATE and GLOW trials (5-year PFS rates of 60% and 52%, respectively). oDespite the shorter follow-up duration, PFS appeared inferior even with FD A + V regimen. (3 years-PFS: 76.5%; 78.8% after censuring deaths due to COVID-19) (11).

A similar trend for a higher PFS rate with an MRD-guided treatment approach was also observed when high-risk patients were considered. Patients with unmutated IGHV showed higher PFS rates with a more prolonged MRD-guided (MDCC and FLAIR trials, 3-year PFS: 90% and 5–5 years-PFS: 94.9%) than a FD treatment (CAPTIVATE trial, 5-year PFS: 53%; AMPLIFY trial, 3-year PFS: 69%). (11, 17, 22, 27)_._ Similarly, higher PFS rates were also reported with an uMRD-guided approach in patients with *TP*53 disruption (MDACC trial, 3-year PFS: 86.1%; SEQUOIA arm D, 2-year PFS: 94%) than a FD treatment (FD-CAPTIVATE trial, 5.5-year PFS: 30%) (12, 17, 21, 28).

These findings suggest that a cBTKi + V MRD-guided approach, which tailors the duration of therapy to the specific dynamics of the disease leads to better outcomes.

### 8.4 Emergence of mutations in BTK and BCL2 and response to retreatment

However, the results obtained with an FD and uMRD approach need to be compared with a longer follow-up and the risk of emergence of mutations. The shorter drug exposure associated with an FD treatment with I + V has been linked to a decreased risk of mutations in BTK, PLCG2, and BCL-2 among patients who experienced disease progression (17, 29, 30), and translates in the high response rates observed in patients of the CAPTIVATE trial who were retreated with single-agent Ib (ORR, 86%) or I + V (7 patients; ORR, 71%) (17). Based on this observation, time-limited regimens combining A or Z plus V are likely to limit the risk of developing BTK or BCL2 mutations.

### 8.5 Safety profile

Treatment-related toxicities with cBTKi + V were consistent with the known AEs associated with each drug when used individually. Notably, the safety profile of these combinations did not reveal any unknown or increased toxicities that would warrant warnings.

Both age and treatment duration had an impact on the rates of toxicities described in patients treated with cBTKi + V. Treatment discontinuations due to AEs were less frequently observed in younger than in older patients (CAPTIVATE trial, 5%; AMPLIFY trial, 7.9%; GLOW trial, 14%) and were also more common in patients receiving prolonged MRD-guided treatment (MDACC trial: 14%; FLAIR trial: 23%; SEQUOIA arm D 25%) (12, 21, 27). As expected, BTKi-related AEs, such as atrial fibrillation and hypertension, were more common in patients receiving I + V than in those treated with A or Z combined with V (AMPLIFY trial, 1%; SEQUOIA armD trial, 3%) (Table 3). Notably, the rates of atrial fibrillation were also higher in patients undergoing longer MRD-guided than FD therapy with I + V (MDACC trial: 10%; FLAIR trial: 13.5% vs. FD-CAPIVATE trial: 4%) (9, 10, 17, 27). No sudden deaths were reported in patients treated with A + V and Z + V, they were rare, 1.6%, in younger patients who received FD I + V in the CAPTIVATE trial and more frequent, 4%, among elderly patients enrolled in the Glow trial (4%) (11, 12, 19). The highest rates of patients with severe neutropenia (51%) and severe infections (22%) were recorded in the MRD-guided study from the MDACC, where treatment was extended up to 36 months (21).

### 8.6 Other V-based treatment options for the front-line treatment of patients with CLL

The FD regimen, including V plus obinutuzumab (VO) regimen for the frontline treatment in both older and younger patients with CLL, was the first and most extensively investigated V-based combination in the CLL14 and CLL13 trials (32, 33). While cBTKi + V, compared with VO, avoids infusion reactions and has a lower risk of tumor lysis syndrome, it carries an increased risk of BTKi-related cardiotoxicity, especially in older patients receiving I. Indirect comparisons between the I + V, A + V, and VO regimens are, not easy because the GLOW, AMPLIFY and CLL13 trials excluded del (17p) patients. Nonetheless, similar -PFS rates were reported with I + V versus V + O in older patients (GLOW trial, 5.5-year PFS: 52%; CLL14 trial, 5-year PFS: 62.6%) and younger patients (FD-CAPTIVATE trial, 4-year PFS: 79%; CLL13 trial, 4-year PFS, 81.8%) (17, 20, 34, 35) but slightly lower with A + V (AMPLIFY, 3-year PFS 69%) (11). Preliminary results from the CLL17 trial (NCT04608318) presented the 2025 ASH congress, showed the non-inferiority of FD I + V regimen or VO combination than continuous ibrutinib.

In addition, the ongoing randomized MAJIC trial (NCT05057494) compares an MRD-guided A + V with the MRD-guided VO regimen.

Combinations that include BTKi + O have been extensively investigated but are not yet approved for the treatment of chronic lymphocytic leukemia (CLL).

Updated data from the CLL13 trials show that adding I to the FD VO (IVO) regimen produces higher uMRD and PFS rates (4-year PFS ≈ 85.5% for the triplet vs. 81.8% for the doublet) but also increases serious toxicity without an overallsurvival advantage (35). Similarly, patients treated with AVO within the AMPLIFY trial experienced very high uMRD rates (67.1%) and sustained PFS (3-year 83.1%) but were hampered by more events of grade 3 or higher (69.4%), in particular severe infections (23.6%) (11).

Impressive data from a study by Davids et al. showed that an MRD-guided therapy using the AVO triplet in an MRD-guided approach achieved a 4-year PFS rate of 77% for patients with *TP*53 disruption (36).

Soumerai et al. reported a 92% rate of responses with uMRD in BM after 8 to 24 cycles of the Z + V + O (BOVEN) triplet, administered in an MRD-guided fashion (37).

Two studies from the Weill Medical College of Cornell University (NCT05650723) and the Italian GIMEMA group (NCT05478512) are investigating in an MRD-guided manner the efficacy and safety of the VO + Z regimen.

Jain et al. demonstrated a 98% rate of uMRD in BM after 13 cycles of the triplet regimen, which included pirtobrutinib + V + O (PVO) (38). In addition, the ongoing CLL18 trial (NCT05057494) will compare the FD pirtobrutinib + V regimen with the MRD-guided Pirtobrutinib + VO triplet.

### 8.7 BTKi combined with novel BCL-2 inhibitors

Several studies are currently investigating whether combining a BTKi with a more selective, new-generation BCL-2 inhibitor results in enhanced effectiveness with reduced toxicity.

With a median follow-up of 19.4 months, preliminary data from the phase I study investigating the second-generation BC-L2 inhibitor sonrotoclax combined with Z demonstrated high rates of uMRD (91%), with no patients experiencing disease progression (39).

Preliminary results presented at the 2024 ASH meeting show a high response rate with another novel BCL-2 inhibitor, lisaftoclax combined with acalabrutinib in relapsed/refractory and treatment-naïve patients with CLL (40).

Promising data from two ongoing trials that are investigating the sonrotoclax and Z combination (NCT06073821, NCT06637501) and the lisaftoclax and A combination (NCT06319456) in previously untreated patients.

### 8.8 BTKi + V for CLL treatment: key points and future perspectives

The strengths of FD BTKi + V combinations should be emphasized. FD therapy combining a covalent BTKi with V is an oral treatment that is easy to manage; it enhances patients’ quality of life by providing a prolonged treatment-free interval, may help minimize long-term toxicities, and limit the risk of the emergence of leukemic clones with resistant mutations of BTK/PLCG2 and BCL-2, allowing, for successful re-treatment. For these reasons, the FD BTKi + V combination should be recommended for patients with mutated IGHV, and without TP53 abnormalities.

While patients under 65–70 years would benefit from either I + V or A + V, more older patients should be treated with A + V given the better safety profile of acalabrutinib (11).

More prolonged PFS is achieved with an MRD-guided treatment than an FD approach, especially in patients with a high-risk genetic profile, such as those harboring TP53 abnormalities and/or complex karyotype (14, 44). This observation suggests a higher proliferative pattern of residual leukemic cells in this patient population. Additional data and a longer follow-up are required to evaluate whether the investigational cBTKi + V regimen delivered in an MRD-guided manner or triplet regimens are more effective than continuous treatment options for high-risk patients (43).

Concerns about safety—especially cardiovascular toxicity in older patients—have led to the recommended I + V primarily for younger and fitter patients (39, 45). These concerns have also contributed, in part, to its limited approval in the United States and other countries.

The availability of second-generation BTK inhibitors, which are more selective and have a favorable toxicity profile, such as A + V and Z + V, will likely extend the use of BTKi + V for elderly and/or comorbid patients. On the other hand, data from the TAILOR trial (NCT05963074), which investigates the benefits of V combined with reduced doses of I, will demonstrate whether this approach can reduce toxicity while maintaining effectiveness.

## 9 Conclusions

The combination of BTKi and V represents a significant advancement in the treatment of CLL. Currently, the FD I + V and A + V regimens are approved and widely used as first-line treatment for patients with CLL and small lymphocytic lymphoma (SLL) (41, 42, 46). As we await further clarification on which patients may benefit most from an FD or MRD-guided treatment or triplet combinations, it is crucial to evaluate and monitor patients closely. This will help ensure the efficacy and safety of the treatment.
