# Supplementary figures and images for: Lights and shades of front-line treatment with covalent BTK inhibitors combined with venetoclax in patients with chronic lymphocytic leukemia
Source: Oncol Rev. 2025 Dec 18;19:1703228. doi: 10.3389/or.2025.1703228 (PMC12756417; doi:10.3389/or.2025.1703228)

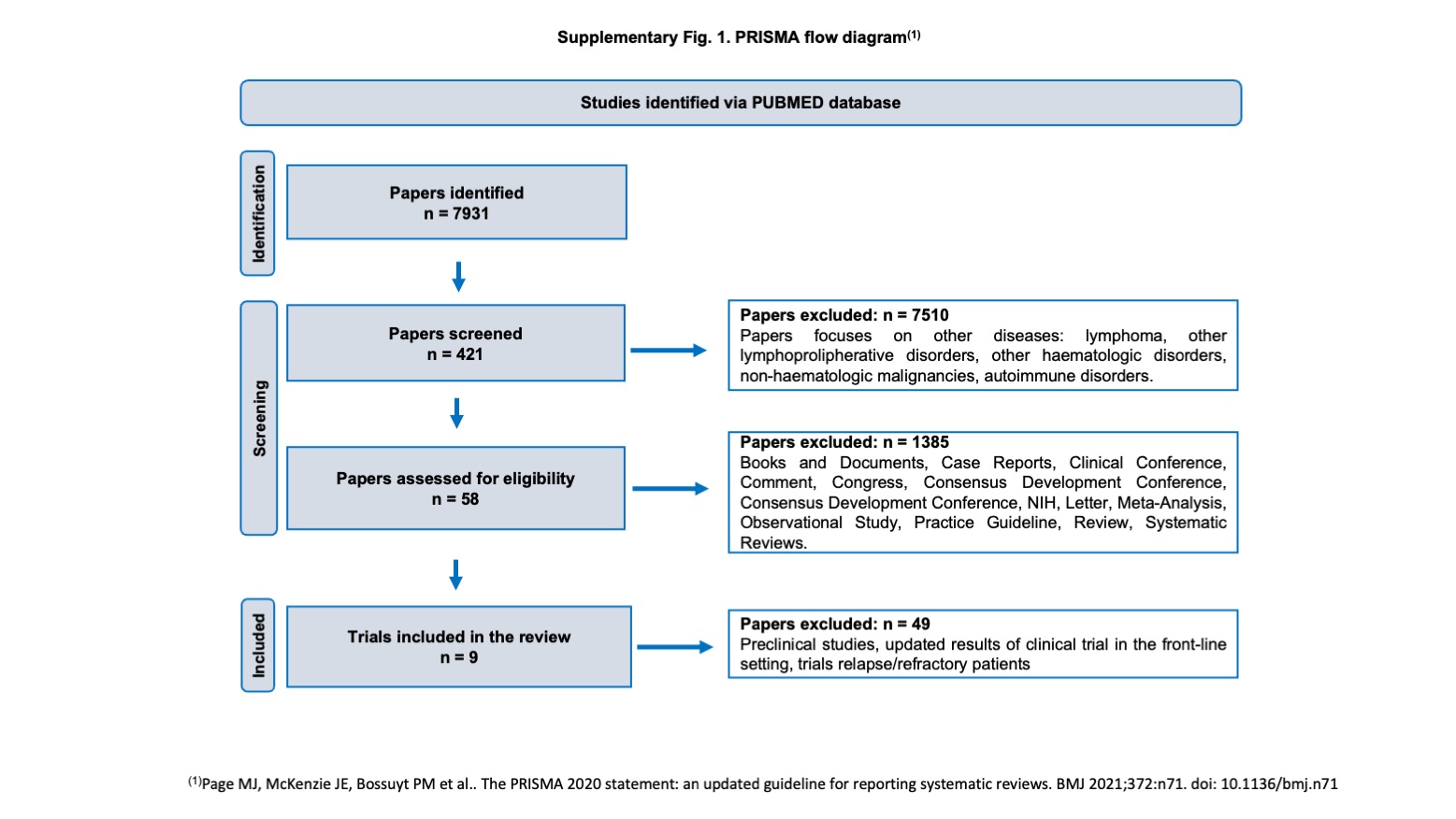

Supplement: Supplementary file 1 [file Image1.JPEG]
